# A probabilistic algorithm for optimising the steady-state diffusional flux into a partially absorbing body

**DOI:** 10.1038/s41598-023-49566-4

**Published:** 2023-12-20

**Authors:** Kyriacos Nicolaou, Bela M. Mulder

**Affiliations:** 1 Institute AMOLF, Science Park 104, 1098XG Amsterdam, The Netherlands; 2 Cell Biology, Neurobiology and Biophysics, Department of Biology, Faculty of Science, Utrecht University, Utrecht, The Netherlands; 3 Institute for Theoretical Physics, Utrecht University, Utrecht, The Netherlands

**Keywords:** Computational biophysics, Computational science

## Abstract

Cells in an aqueous environment absorb diffusing nutrient molecules through nanoscale protein channels in their outer membranes. Assuming that there are constraints on the number of such channels a cell can produce, we ask the question: given a nondepleting source of nutrients, what is the optimal distribution of these channels over the cell surface? We coarse-grain this problem, phrasing it as a diffusion problem with position-dependent Robin boundary conditions on the surface. The aim is to maximize the steady-state total flux through the partially absorbing surface under an integral constraint on the local reactivities. We develop an algorithm to tackle this problem that uses the stored and processed results of a particle-based simulation with reflective boundary conditions to a posteriori estimate absorption flux at essentially negligible additional computational cost. We validate the algorithm against a few cases for which analytical or semi-analytical results are available. We apply it to two examples: a spherical cell in the presence of a point source and a spheroidal cell with an isotropic source at infinity. In the former case, there is a significant gain relative to the homogeneous case, while in the latter case the gain is only $1\%$.

## Introduction

Many aquatic organisms, such as bacteria or simple multicellular assemblies, such as green algae, rely on nutrients dissolved in their watery environment. These nutrients are taken up by protein assemblies at the cell surface that actively or passively facilitate their transfer to the cell interior^1,2^. Given that the cell will only produce a finite number of these transporter complexes, this raises an interesting optimization problem: given a specific source of nutrients and the shape of the organism, what is the optimal distribution of the transporters? In general, this is clearly a challenging problem as a multitude of complexities are involved, ranging from the role of the motion of the organism, flows in the environment, and possibly competition with other organisms. Here, we wish to take a first step and pare this problem down to a simple and mathematically tractable setting. We therefore ignore all dynamical aspects and focus on the case of a single isolated organism suspended at rest in an infinite reservoir without any fluid flow, ruling out any convective effects. We also assume that the dimensions of the organisms (typically larger than a few micrometers) are large with respect to the dimensions of the uptake channels (typically on the nanometer scale). This allows us to model the uptake through Robin-type boundary conditions on the diffusion with the reactivity, the flux of absorbing particles per unit area in units of the concentration, proportional to the local density of transporter complexes. In this setting, we attempt to answer the following question: What is the distribution of reactivities that maximizes the overall steady-state flux of nutrient particles absorbed by the organism?

To answer this question, we clearly need an efficient way to accurately evaluate the steady-state flux both for different geometries and different distributions of reactivities. Very recently, several such techniques have been developed for spherical bodies, where^3^ addresses the case of arbitrary distributions of reactivities, while^4,5^ focus on spherical traps. For generic geometries, the most widely used method to deal with this type of boundary problem arguably remains the finite element method (FEM, see, e.g.^6^). However, FEM has a number of drawbacks. First of all, it discretizes space and thus requires a mesh of varying homogeneity for every geometry. Second, it requires an outer boundary and can thus only approximately deal with boundary conditions at infinity. Moreover, as the spatial extent of the depletion layer surrounding a partially absorbing body will depend on the total reactivity, which we would like to vary, one would ideally need to adapt the dimensions of the outer boundary on a case-by-case basis. Finally, and in the light of our problem most importantly, it can only deal with a specific boundary condition at the time, which is disadvantageous for any numerical optimization process that requires many evaluations of the target function for different parameters.

Problems with reactive boundary conditions of Robin type abound in the field of biomolecular interactions. In addition to absorbing molecules through importer channels, cells sense their biochemical environment through receptor molecules embedded in their membranes^7^. The binding of ligands to these receptors is a rate-limited step, which macroscopically leads to this type of boundary conditions. Over the years, several stochastic techniques have been developed to simulate the behavior of such systems using Brownian motion and concepts from the probabilistic interpretation of the diffusion equation^8–12^.

Here we combine a number of elements from these latter techniques to propose an alternative approach to the problem of partially absorbing convex bodies with inhomogeneous reactivity distributions. This approach has a number of advantages, respectively, offsetting each of the disadvantages of FEM mentioned above. First, it does not require any spatial discretization. Only in a small neighborhood of the partially absorbing body is a finite time step space-continuous Brownian process required, which introduces a weak but spatially uniform step size dependence. Second, it leverages results from potential theory to deal with the boundary conditions at infinity exactly. Finally, and most crucially, we build on the seminal results of Filoche, Sapoval, and Grebenkov^3,13,14^, who showed that knowledge of the so-called Brownian self-transport operator, which encodes the first-passage probabilities that a random walk starting from a surface lands elsewhere on the surface, can be used to solve partial absorption problems. In its original form^13,14^ it was applied to 2D surfaces with complex irregular boundaries with uniform reactivity, modeling resistive electrodes, using a fully discretized random walk model. In the more recent development, Grebenkov^3^ showed how the so-called Dirichlet-to-Neumann operator, essentially the continuum limit of the Brownian self-propagator, can be used to formally solve the Robin boundary problem. The latter quantity can be spectrally approximated, which, for technical reasons for now, limits its application to spherical geometries. Our approach in a sense interpolates between these two earlier approaches: we use a *continuous* space random walk to sample the Brownian self-propagator between *discretized* domains on the surface in a, for a given shape of the body, *single* set of simulations with * reflective* boundary conditions. This quantity then allows us to build a discrete-state Markov model with which we can a posteriori evaluate the flux for any distribution of local reactivities at essentially negligible additional computational cost. This latter feature, which we dub “Fly now, pay later”, more than offsets the additional computational cost of a stochastic simulation with respect to FEM on a single shot basis and enables us to efficiently perform the required optimizations.

## Results

## Problem formulation

The problem we pose is the following: for a given nonspherical body, what is the optimal distribution of the local reactivities over its surface such that the steady-state diffusional flux through the surface is maximized, under the constraint that the total reactivity is fixed. In order to avoid some minor technical details, we limit ourselves to convex bodies. We will consider two cases, the first in which the density of diffusing particles is fixed to a constant value $n_{\infty }$ in the bulk far from the organism and the second in which the diffusing particles are emitted from a constant rate source at some distance from the body. In the first case, the steady-state density of particles $n({\textbf{r}})$ satisfies the homogeneous Laplace equation

1 $$\begin{aligned} \Delta n\left( {\textbf{r}}\right) =0 \end{aligned}$$

with boundary condition at infinity

2 $$\begin{aligned} n\left( \left| {\textbf{r}}\right| \rightarrow \infty \right) =n_{\infty }, \end{aligned}$$

while in the second case, the density satisfies the inhomogeneous equation

3 $$\begin{aligned} D \Delta n\left( {\textbf{r}}\right) + \Phi _S \delta ({\textbf{r}}-{\textbf{s}})=0, \end{aligned}$$

where *D* is the diffusion constant, and ${\textbf{s}}$ the location of the source with production rate $\Phi _S$.

At the surface of the convex body *A* we have the Robin boundary condition

4 $$\begin{aligned} D\mathbf {\nabla }n\left( {\textbf{R}}\right) \cdot \hat{\mathbf {\omega }}\left( {\textbf{R}}\right) =k\left( {\textbf{R}}\right) n\left( {\textbf{R}}\right) ,\;{\textbf{R}}\in \partial A, \end{aligned}$$

where $\mathbf {\hat{\omega }}\left( {\textbf{R}}\right)$ is the unit normal *outward to the absorbing body* at ${\textbf{R}}$ and $k\left( {\textbf{R}}\right)$ the local reactivity. The total absorptive flux through the boundary is then obtained by integrating

5 $$\begin{aligned} \Phi _{A}\left[ k \right] =D\int _{\partial A}d\sigma _{A}\left( {\textbf{R}}\right) \,\mathbf {\nabla }n\left( {\textbf{R}}\right) \cdot \hat{\mathbf {\omega }}\left( {\textbf{R}}\right) , \end{aligned}$$

where $d\sigma _{A}\left( {\textbf{R}}\right)$ is the infinitesimal element of surface area on the boundary $\partial A$. Our goal is to determine the distribution of the local reactivities $k_\text {max}$ that maximizes the total flux

6 $$\begin{aligned} k_\text {max}={\text {argmax}}_{k} \Phi _{A}\left[ k \right] , \end{aligned}$$

under the constraint that the *mean reactivity*

7 $$\begin{aligned} k_{\text {ave}} = \frac{\int _{\partial A} d\sigma _{A}({\textbf{R}}) k({\textbf{R}})}{\int _{\partial A} d\sigma _{A}({\textbf{R}})}, \end{aligned}$$

is fixed. This choice of constraint divides out the trivial dependency on the total surface area and allows us to focus on differences caused by differences in shape.

We will approach this problem in a probabilistic setting. Our starting point is a direct generalization of a well-known result of probabilistic potential theory^15^. Consider a convex set *B* such that our target body *A* is fully enclosed by *B*. To reach *A* and be absorbed, a diffusing particle originating at infinity must first have entered *B* through a point ${\textbf{R}}^{\prime }$ on its surface $\partial B$. From this intermediate position, the particle has a probability $P_{A}[k]\left( {\textbf{R}}^{\prime }\right)$ of ultimately being absorbed. Alternatively, with probability $1-P_{A}[k]\left( {\textbf{R}}^{\prime }\right)$ it “escapes” to infinity. This observation allows us to write the flux through *A* as the following surface integral over *B*

8 $$\begin{aligned} \Phi _{A}[k] =\int _{\partial B}d\sigma _{B}\left( {\textbf{R}}^{\prime }\right) \,P_{A}[k]\left( {\textbf{R}}^{\prime }\right) \varphi _{B}\left( {\textbf{R}}^{\prime }\right) , \end{aligned}$$

where $\varphi _{B}\left( {\textbf{R}}^{\prime }\right)$ is the flux density of particles arriving at location ${\textbf{R}}^{\prime }\in \partial B$ *without previously having entered* *B* (See Supplementary Information Fig. 1). Note that the latter density is obtained simply by solving the analogous diffusion problem for the body *B* assuming that it is a perfect absorber. Now consider the special case that $B=B\left( 0,R_{B}\right)$ is a sphere of radius $R_{B}$ whose origin we can take, without loss of generality, to be the origin of the centroid of *A*. In this case, the first passage flux through *B* is simply given by

9 $$\begin{aligned} \varphi _{B}\left( {\textbf{R}}^{\prime }\right) = \frac{D}{R_{B}}n_{\infty } \equiv \frac{1}{4\pi R_{B}^{2}}\Phi _{B} \end{aligned}$$

Thus we can write

10 $$\begin{aligned} \Phi _{A}[k] =\left( \frac{1}{4\pi R_{B}^{2}}\int _{\partial B}d\sigma _{B}\left( {\textbf{R}} ^{\prime }\right) \,P_{A}[k]\left( {\textbf{R}}^{\prime }\right) \right) \Phi _{B} \equiv P_{A}(\partial B) \Phi _{B} \end{aligned}$$

We recognize the first factor between parentheses as the surface averaged value of the probability that a particle launched from the surface of the sphere *B* is absorbed by *A*, which we denote by $P_{A}(\partial B)$. In the case of a point source, we can simply directly calculate

11 $$\begin{aligned} \Phi _{A}[k] = P_{A}[k]\left( {\textbf{s}}\right) \Phi _{S} \end{aligned}$$

The challenge we face is to estimate the probabilities $P_{A}(\partial B)$ and $P_{A}[k]\left( {\textbf{s}}\right)$ efficiently for a range of reactivity distributions *k*.

## Computational approach

Our computational approach has three components. The first component, the ’fly-now part,’ is a customized stochastic simulation of diffusing particles that are reflected at the boundary of the body in question. From a starting position outside we track where (if at all) the particle hits the surface of the body until it inevitably escapes to infinity. From this data we derive a set of transition probabilities between the relevant states. These probabilities are used in the second component, the ‘pay-later’ part, to evaluate a posteriori the relevant local and global absorption probabilities needed to evaluate the steady-state flux in the partially absorbing case. Finally, in the third component, we perform optimization of the total flux over the distribution of the local reactivities. Refer to the Methods section for more details of these three components.

### Validation

In order to validate our algorithm, we need to compare it to independent results. To our knowledge, the problem posed by Eqs. (1), (2) and (4) only admits a closed form solution for finite nonzero reactivity when $A=B\left( 0,R\right)$, i.e. a sphere with radius *R*, and then only when the reactivity is homogeneous on the surface, $k\left( {\textbf{R}}\right) =k$, so that the problem is spherically symmetric (see e.g.^16,17^). Indeed, the only spherically symmetric solution to the Laplace equation which is bounded on ${\mathbb {R}}^{3}/A$ is given by

12 $$\begin{aligned} n\left( r\right) \propto c_{0}+c_{-1}\frac{1}{r}. \end{aligned}$$

The boundary conditions then dictate that the solution is

13 $$\begin{aligned} n\left( r\right) =n_{\infty }\left( 1-\left( \frac{kR/D}{1+kR/D}\right) \frac{R}{r}\right) , \end{aligned}$$

and the total flux follows to be

14 $$\begin{aligned} \Phi _{A}\left[ k\right] =4\pi D R n_{\infty }\left( \frac{kR/D}{1+kR/D}\right) \end{aligned}$$

In the limit that the reactivity diverges, $k \rightarrow \infty$, the body becomes fully absorbent, and we recover the well known result $\Phi _{A}\left[ \infty \right] =4\pi D R n_{\infty }$.

Throughout, in presenting our results, we will take a sphere $U=B(0,1)$ with radius $R_0=1$ as our reference, which fixes our unit of length, and take the surface area of all different shapes we consider equal to that of the reference sphere, i.e. $|\partial A|=4\pi$. Moreover, we use the dimensionless mean reactivity $K\equiv k_{\text {ave}} R_0/D$ to set the numerical value of the global constraint on the reactivities.

**Figure 1:**
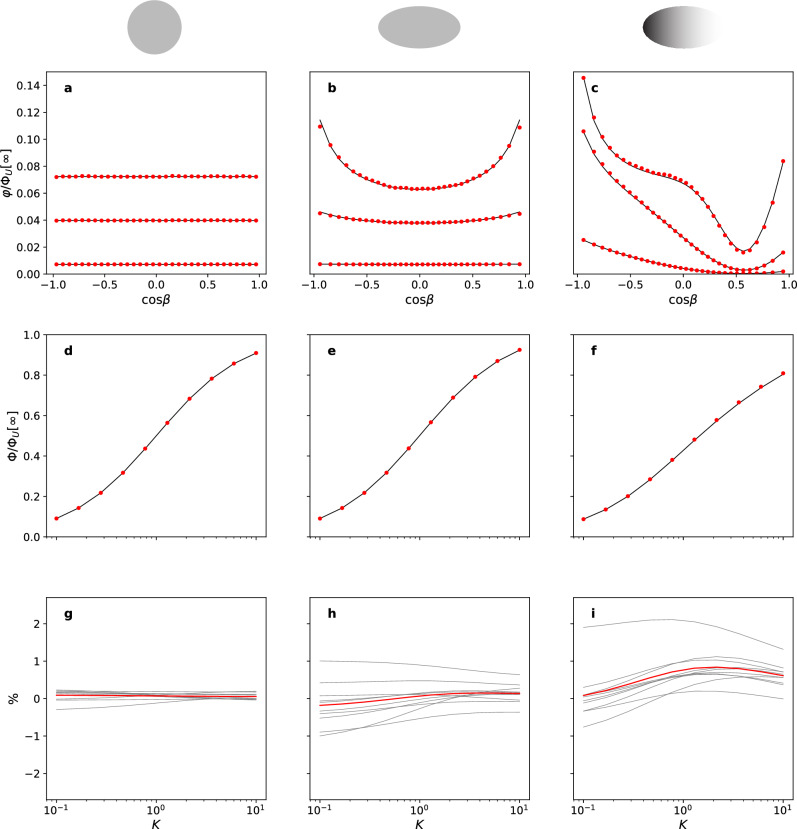
Validation results for, from left to right, a homogeneously partially absorbing sphere, a homogeneously partially absorbing spheroid with aspect ratio $\gamma =3$, a spheroid with $\gamma =3$ with an inhomogeneous local reactivity of the form SI Eq. (49) with parameters $\kappa _0 = 1.102$, $\kappa _1= -1.827$, and $\kappa _2 = 1.049$. The longitudinal position along the body is measured in terms of the polar ellipsoidal angle $\beta$ (See SI Eq. (47)), which for the spherical case coincides with the usual polar angle. Throughout results from our approach are in red, while the black solid lines are the reference results, exact in the case of a sphere, and obtained using the Piazza–Grebenkov formalism for the cases with the spheroid. Panels (**a, b, c**): results for local steady-state flux in units of the total flux $\Phi _{U}[\infty ]$ through a perfectly absorbing sphere of unit radius as a function of a scaled coordinate along the long axis of the body for three values of the mean reactivity: from the bottom to the top $K=0.1$, $K=1$ and $K=10$. Panels (**d, e, f**): results for the total steady-state flux as a function of $0.1 \le K \le 10$. Panels (**g, h, i**): relative error in the global flux with respect to the reference data in percentages. The gray lines are data taken with $N=10^5$ particles, and the red line is the result of pooling these subsamples. Throughout, we used the Brownian step size $\lambda = 0.001$ and $N=10^6$ particles. The number of discrete domains used to build the Markov model was $M=32$.

In the leftmost column of Fig. 1 we show the results of our algorithm for the case of the sphere, respectively, for the local flux, the total flux normalized to that of a totally absorbing sphere of the same radius, and the estimated error compared to the exact results.

When nonspherical bodies are considered, the most tractable types are naturally ellipsoids. The Laplace equation is separable in the ellipsoidal coordinate system^18^. As the surface of an ellipsoid is described by a constant value of the first ellipsoidal coordinate, both the Dirchlet problem with a uniform surface value and the zero-flux von Neumann problem admit closed-form solutions. However, in the generic Robin boundary condition case with nonzero reactivity, the presence of a position-dependent geometrical factor in the normal derivative breaks the separability of the boundary conditions, precluding a closed-form solution in this coordinate system. Fortunately, Piazza and Grebenkov (PG) have recently presented a rapidly converging technique for approximating the exact solutions on arbitrary axisymmetric convex bodies^19^, which can also be generalized to deal with inhomogeneous distributions of local reactivity constants. Here we use their approach to generate high-quality approximate solutions, to which we can compare our stochastic results for the case of prolate spheroids. Relevant details on the description of the spheroids, the construction of two-parameter inhomogeneous reactivity distributions, and the application of the PG technique are collected in Supplementary Information (SI) 5. In the middle column of Fig. 1 we show the results for a spheroid with aspect ratio $\gamma =c/a=3$ with a homogeneous distribution of local reactivities, while in the right column we show the same spheroid, but now with an inhomogeneous local rate distribution.

A few salient observations follow from these results. First of all, the quantitative agreement with the reference values is more than satisfactory. Using subsampling of the relative error, shown in the bottom row of Fig. 1, to look at the behavior of the error as function of the sample size *N*, we do see that the estimated error in the error increases as we go from sphere to homogeneous ellipsoid to inhomogeneous ellipsoid. The mean error, however, does not deviate by more than $0.5\%$ from zero, suggesting that systematic errors are small at best. At the same time it appears that the discretisation employed in our stochastic algorithm (here $M=32$) is sufficient to capture the local flux variations even in the most inhomogeneous cases considered. From a physical perspective the most prominent result is that the specifics of the non-spherical geometry or the inhomogeneity of the rate distributions are only revealed at higher mean reactivities. Looking at the local flux profiles shown in the top row of Fig. 1, we see that only for the highest value $K=10$ the homogeneous ellipsoid shows the expected increase in local flux at the more curved poles and the inhomogeneous ellipsoid closely tracks the imposed reactivity profile. For the homogeneous high-reactivity case, these latter results are also similar to the recent results in^20^ for the perfectly absorbing case $K \rightarrow \infty$. The underlying common cause of these observations is that at low mean reactivities the particles effectively diffusively explore the whole surface, thereby averaging out any local features. This is evident for the flux profiles for the smallest $K=0.1$, which in all cases are almost flat, even when the imposed reactivity profile is strongly varying.

### Applications

**Figure 2:**
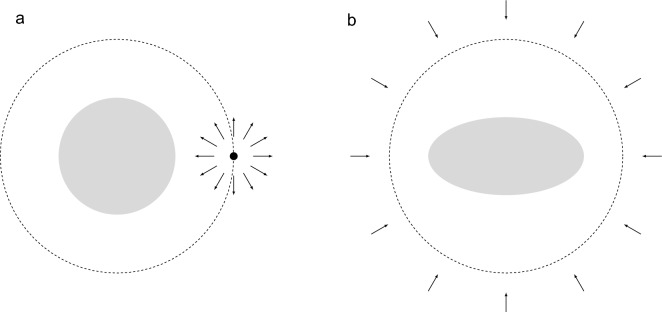
(**a**) A spherical body in the presence of a point source of constant rate. (**b**) A uniaxial nonspherical body with an isotropic bulk source at infinity modelled by a fixed concentration.

Having validated our computational framework, we now consider its application to the two scenarios illustrated in Fig. 2. In both cases, we address the question of the optimal distribution of reactivities over the surface of the body that maximizes the steady-state absorptive flux, under the constraint that the total reactivity is fixed.

#### Sphere with point source

**Figure 3:**
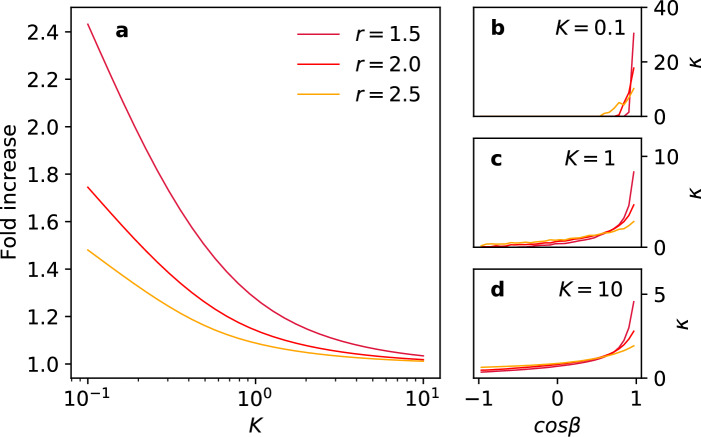
Panel (**a**): Fold increase of the total flux with respect to a homogeneous sphere as function of the mean reactivity *K*, for a point source at three different distances *r* from the sphere on the *z*-axis of the reference frame. Panels (**c, d, e**): the optimal local reactivity $\kappa (\cos \beta )$ as function of position parametrized by the angle $\beta$, for three values of the mean reactivity.

Although from a biological we are primarily interested in our original question of a nonspherical organism in a uniform nutrient bath, there are many situations in which cells or organisms are exposed to nonuniform gradients of diffusive substances. The typical response to adapt to these conditions is either a static or dynamic rearrangement of receptor proteins to optimize chemoperception (for a recent perspective, see^21^). Examples range from bacteria-chasing neutrophils^22^, chemotropism in fungi^23^, to the distribution of receptors on neural synapses^24^. Therefore, we also chose to model the most extreme case of an anisotropic source, viz. a point source. Point sources are readily accommodated into our framework and can also be used as a basis to build more complex source distributions. Our results in this case are summarized in Fig. 3.

A number of readily understood features stand out in these results. The first is that the relative gain in total flux of polarizing the reactivity distributions towards the source is most prominent at lower mean reactivities. In this case, the higher probability of first contact with the sphere of the diffusing particles in the neighborhood of the point closest to the source is optimally exploited. As the overall reactivity increases, the relative gain with respect to the homogeneous case decreases and, in fact, tends to unity in the limit of total reactivity $K\rightarrow \infty$. Consistent with this trend, the lower the mean reactivity, the more polarized the optimal reactivity distributions are. The second is that the relative gain decreases as the source is moved farther away from the sphere, which depolarizes the first-passage distribution of the particles on the sphere. In fact, one expects that in the limit $r\rightarrow \infty$ the diffuse source limit is reached, in which the optimal distribution by symmetry must be homogeneous.

#### Uniaxial bodies with a bulk source

Our second application refers back to the original motivation for this work, namely the question of whether an aquatic organism can maximize its nutrient uptake by optimally distributing its uptake channels over its surface. We first choose to model the organism as a prolate spheroid and assume that the source of nutrients is diffuse and represented by a constant concentration at infinity.

Considering first the case of a perfect absorber or its electrostatic analog a grounded perfect conductor, it is known that the local flux or equivalently the normal electric field, and hence the induced charge, is maximal at the more highly curved poles (for a classical reference, see^25^). On the basis of this fact, one could naively assume that in the partially absorbing case it would therefore pay off to concentrate the constrained absorptive capacity in the polar regions.

**Figure 4:**
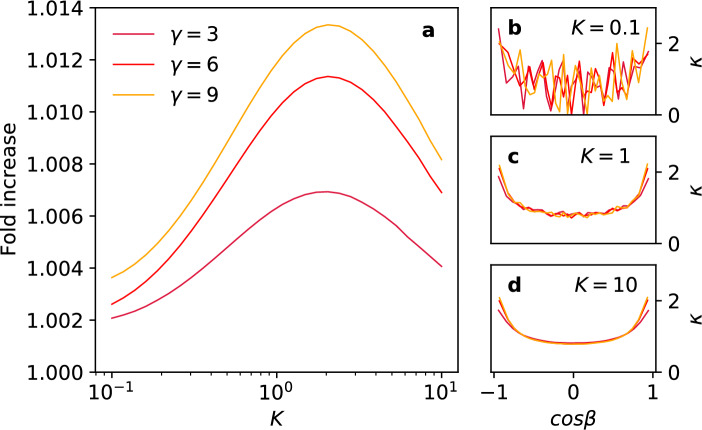
Panel (**a**): Fold increase of the total flux with respect to a homogeneously absorbing spheroid as function of the mean reactivity *K*, for spheroids with increasing aspect ratio $\gamma$. Panels **(b, c, d**): the optimal local reactivity $\kappa (\cos \beta )$ as a function of the position parametrized by the polar spheroidal angle $\beta$, for three values of the mean reactivity.

The results we obtained are shown in Fig.  4. We do indeed see that across the board the total flux is enhanced with respect to the homogeneous case by a distribution that is maximal at the poles, and that this effect becomes stronger for the more elongated ellipsoids. However, the most striking and admittedly initially disappointing result is that the magnitude of the gain achieved is only of the order $1\%$ at best. Although the results are systematic and as far as we can judge significant given our error estimates, we tried to independently validate the nonnull result on the gain by applying the semianalytical PG technique to this case, which allows us to optimize over a limited set of reactivity distributions. The results presented in SI 5.4 show that also in that case a small nonzero gain is predicted for a distribution focused to the poles. In order to see to what extent the specific shape of the elongated body plays a role, and at the same time highlighting the ability of our approach to deal with different shapes, we repeated these calculations for spherocylinders, convex bodies consisting of a right circular cylinder capped at both ends by a half-sphere. The results are presented in SI 6 and show a similar outcome to the spheroid case, viz. a gain of $~1\%$ for reactivity distributions focused on the spherical caps. The only difference appears to be that the dependence on the length of the cylindrical segment quickly saturates, indicative of the fact that the flux becomes localized to a finite region close to the spherical caps.

Our intuitive understanding of the result of the limited enhancement of the total flux with respect to the homogeneous case is as follows: for small values of the mean reactivity *K* the probability of absorption is so low that multiple attempts are required to absorb, if at all. In this case, the particles explore the entire surface area, effectively averaging out variations in the local reactivity. This is also consistent with earlier results on reflecting spheres with circular absorbing patches^26–29^ that show that for low surface coverage of traps, corresponding to the case of low mean reactivity, the spatial distribution of the traps becomes irrelevant. We also note that for low mean reactivities, the optimal distribution appears rather noisy (see Fig. 4b). This is caused by the inevitable finite sampling errors in the reflective transition probabilities. Effectively, one needs larger-order products of these probabilities that magnify relative errors to determine the local fluxes that enter the optimization procedure.

At the other extreme of very strong absorption, $K\rightarrow \infty$, the effects of partial absorption saturate [cf. the result for the sphere Eq. (14)] and there is nothing to gain by redistributing. Therefore, gain can occur only at intermediate values of *K*. However, in this case, the gain achieved by shifting the distribution of the reactivities towards the more curved poles, which by construction comprise but a small fraction of the total surface area, appears to be offset by the loss incurred by having less absorption take place over the larger fraction of the surface that is less curved. To bolster this intuition we have considered an extremely simplified toy model: two homogeneously partially absorbing spheres of unequal radius, and hence unequal curvature, a large distance apart, and hence not influencing each other’s absorption. In this case, the trade-offs in redistributing a fixed total reactivity over the two spheres can be analytically calculated. The details of this calculation are given in SI 7. Interestingly, for this situation the global maximum in the gain, obtained by simultaneously optimizing over the distribution of rates, the mean reactivity, and the relative radius, turns out to be just over $5\%$. Our expectation is that if the two spheres are moved closer to each other and the incoming flux field at each sphere becomes distorted by the presence of the other sphere, thus resembling more closely the case of a single connected body with regions of different curvature, this maximum gain can only decrease as the more stronger absorbing smaller sphere can no longer maximally exploit the available incoming flux which is partially diverted to the less-absorbing other sphere.

## Discussion

The goal we hoped to reach in this work was to understand whether a non-spherical aquatic organism could, in principle, improve its nutrient uptake by concentrating its importer channels on the more strongly curved parts of its surface. Although our final result, which showed that the gain in doing so is likely too small to confer a significant advantage, turned out ‘poor’, our journey towards this goal was ‘full of adventure, full of discovery’, as C. P. Cavafy famously described as a metaphor for human endeavors in general: without this ‘Ithaka’ in mind, we would not have set out on this journey (p. 36)^30^. The method we have developed for treating problems involving Robin boundary conditions has many potential applications. To that end, we note that it can be readily extended in several directions. First of all, although for conceptual simplicity we limited ourselves to a single convex body, it is clear that the stochastic algorithm would also work for multiple and not necessarily convex bodies, provided that the radii of curvature and the mutual distances involved are large with respect to the Brownian step size $\lambda$. Next, although here we focused on the steady state, the algorithm can also be generalized to work in the time domain. For spheres the hitting time and place distributions, both for starting points inside and outside the sphere, are analytically known^31^. Sampling from these distributions then allows the timing of both the WoS moves and the return to the enclosing sphere moves to be included. As the Brownian simulation close to the bodies is already in the time domain, this would allow the full temporal evolution to be tracked and stored. Rather than simply determining the transition probabilities between reflection events in the discrete set of surface domains, as we did here, one would then, of course, have to determine the full transition time distributions involved. This is a straightforward, albeit potentially computationally challenging procedure, whose feasibility needs to be explored. Here, the idea recently promoted by Grebenkov^32^ to consider the so-called local boundary time in this context may also be relevant. Finally, we only discussed the case of absorption. However, it is easy to extend our approach to the case of transient binding. This latter extension is e.g. important in the context of the ubiquitous ligand-receptor interactions that play a major role in inter- and intracellular signalling and sensing. In the simplest case, this would involve the specification of an unbinding rate that governs the Poissonian dissociation of ligands from the receptors. An exciting (pun intended) application for these ideas would e.g. be the study of the influence of the spatial distribution of receptor complexes on synapses on neuronal signaling^24^.

## Methods

### Stochastic reflective boundary simulation

In the simulation particles are launched one-by-one either form a random point on the boundary of the *enclosing sphere* *B* that surrounds *A*, for the case of a fixed density at infinity, or from the fixed source, which by construction is placed on the boundary of the enclosing sphere. The particles are then propagated using the walk-on-spheres (WoS) algorithm^33^, in which they isotropically sample the surface of the largest possible sphere that does not intersect with the absorbing body. If after such a move they end up outside the reference sphere, they either escape to infinity and their run ends or they are placed back on the reference sphere by sampling the known distribution for the first passage location of a diffusing particle landing on a sphere, a technique first described in^34^. If they arrive at a location within a boundary layer of points closer to the surface than a fixed small distance $d_{*}$, the simulation switches to a standard finite-time step Brownian diffusion simulation. If a particle ends up at a location inside *A* it is reflected back into the boundary layer. As soon as a particle exits the boundary layer, it switches back to the WoS algorithm. Ultimately, all individual runs end by escape to infinity. More technical details on the algorithm are relegated to SI 1.

**Figure 5:**
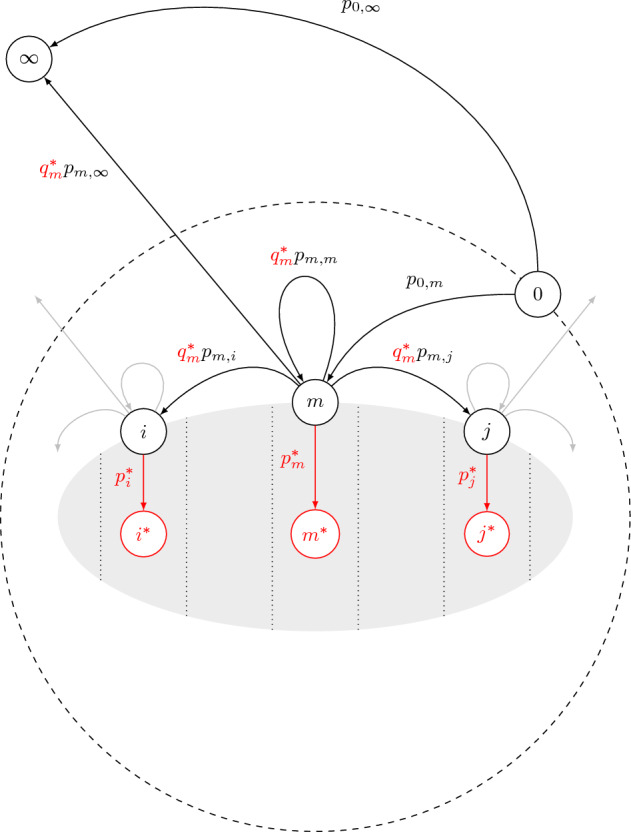
Schematic showing the states and transitions used in the calculation of the absorption probabilities. In black states considered in the reflective boundary simulation: 0 is the starting state, *i*, *m* and *j* states corresponding to a reflection event in a specific discretized domain on the surface, indicated by the dotted boundaries, and $\infty$ the absorbing state at infinity. The black arrows denote transitions between states in the reflective simulation, with their associated transition probabilities $p_{s,s'}$. The gray arrows emanating from states *i* and *j* generically represent transitions to other possible states. The additional absorbing states associated with each domain are indicated in red, with the transitions toward them labeled by the corresponding absorption probability ${p_s^*}$. Note that in the Markov model that includes absorption, the “bare” transition probabilities $p_{i,j}$ between the surface states obtained from the reflective simulation are “dressed” by the probability of nonabsorption ${q_i^{*}=1-p_i^{*}}$.

We divide the surface $\partial A$ of the body into *M* equal-area domains organized linearly along an axis, which in the specific cases considered below coincides with a symmetry axis of the body. In the simulation we now track the transitions between the following states: Being in the starting state 0, being reflected from the surface in domain *m*, and escaping to infinity $\infty$. Accumulating $N_{i,j}$, the number of times a transition occurs between states *i* and *j*, then allows us to estimate the transition probability matrix $p_{i,j}$, which forms the basis for the absorption calculations in the next component. The states and the transitions between them are illustrated in Fig. 5.

The three main parameters of the diffusion algorithm are the radius $R_B$ of the enclosing sphere, the distance $d_*$ from the surface below which the algorithm performs a Brownian simulation, and the step size $\lambda =\sqrt{4D\Delta t}$ of the Brownian process within the boundary layer. Although in principle the results should be insensitive to the choice of $R_B$, a smaller value reduces the number of walk-on-spheres steps. In practice, we use the value $R_{B}=1.1 \times \text {half the maximal dimension of the convex body}$. For the Brownian step size, we use $\lambda =0.001$ in units where the dimensions of the convex body are ${\mathcal {O}}(1)$ and the thickness of the boundary layer is then taken to be $d_*=4 \lambda$. These choices balance the need for sufficient spatial resolution to resolve the geometry of the surface with the number of computationally costly Brownian steps, while still allowing sufficient opportunity to probe collisions with the surface. As a final tweak, we subtract $\delta =2 \lambda$ from the distance calculation in the walk-on-spheres algorithm, so that a WOS jump cannot bring the particle closer than half a boundary layer away from the surface, which avoids a slight undersampling of collisions. Finally, the number of particles simulated, which in the results presented here is $N_{\text {try}}=10^6$.

### Evaluating the steady-state absorption

In order to evaluate the probability of a particle being absorbed at a reflection event, we use the expression derived by Singer et al.^35^ constructed so that the finite time step result matches the macroscopic boundary condition for a flat interface bounding a half-space,

15 $$\begin{aligned} P_{\textrm{abs}}[k]({\textbf{R}})=\sqrt{\frac{\pi }{D}}k({\textbf{R}})\sqrt{\Delta t}. \end{aligned}$$

In SI 3 we recapitulate the relevant derivation, explicitly showing that, as Singer et al. already surmised, this expression also holds for a smooth curved surface whose radii of curvature are large compared to the Brownian step size.

Armed with this expression, we introduce reactivity distributions that take on constant values on the discretized surface domains introduced above. Within each domain labeled with index *i* we have a given value $k_i$ for the local reactivity, to which we associate the absorption probability $p_i^*$ calculated using Eq. (15), and its conjugate $q_i^*=1-p_i^*$. To each domain we associate an absorbing state $i^*$ to which the particle transitions after an absorption event. Putting all these elements together then yields a Markov chain model, in which the “bare” reflective transition probabilities between the surface states and the escape to infinity state need to be “dressed” by the corresponding conjugate probability $q_i^*$ of not being absorbed at the source domain. The additional states and their associated transition rates are illustrated in Figure 5.

It is now a standard exercise in Markov chain theory to obtain the steady-state fluxes into the absorbing states within the model. The details of this calculation are relegated to SI 4. The upshot is that we can leverage the data obtained from the stochastic simulation to obtain the absorption fluxes for any arbitrary distribution of local reactivity at the essentially negligible additional computational cost required for the matrix operations involved.

### Optimisation of the total flux

In this last component, we use the machinery of the previous two components to optimize the total flux. To that end, we consider discretized distributions of local reactivities $k_i$ as introduced above under the constraint

16 $$\begin{aligned} k_{\text {ave}}= \frac{1}{M}\sum _{i=1}^{M} k_i \end{aligned}$$

which fixes the mean reactivity ${\bar{k}}$. This *M*-dimensional optimization problem we tackle by first generating a initial candidate distribution, which is subsequently improved upon by employing a Sequential Least Squares Programming algorithm^36,37^. For obtaining the candidate initial distribution, we have two alternative approaches. The first is a brute-force search achieved by sampling the Dirichlet distribution of the polytope implied by the constraint (16). The second uses distributions sampled from a small two-dimensional parameter domain spanned by two orthogonal polynomials on the surface. We describe this in more detail in SI 5.4, where we apply it to the case of spheroidal bodies.

### Supplementary Information

Supplementary Information.

## Data Availability

The simulation program and data analysis scripts used in the current study are available for download at https://github.com/kyriacosn/Fly-Now-Pay-Later-An-algorithm-for-diffusion-absorption-calculations.
