# Supplementary material for: A probabilistic algorithm for optimising the steady-state diffusional flux into a partially absorbing body
Source: Sci Rep. 2023 Dec 20;13:22815. doi: 10.1038/s41598-023-49566-4 (PMC10739912; doi:10.1038/s41598-023-49566-4)
Supplement: Supplementary file 1 — Supplementary Information. [file 41598_2023_49566_MOESM1_ESM.pdf]

Supplementary information to: A probablistic algorithm for optimising the steady-state diffusional flux into a partially absorbing body

Kyriacos Nicolaou[1,2] **and Bela M. Mulder**[1,2,3,*]

[1]Institute AMOLF, Science Park 104, 1098XG Amsterdam, the Netherlands
[2]Cell Biology, Neurobiology and Biophysics, Department of Biology, Utrecht University, Utrecht, the Netherlands
[3]Institute for Theoretical Physics, Utrecht University
[*]mulder@amolf.nl

ABSTRACT

These supplementary notes bring together all the more technical material used in "A probablistic algorithm for optimising the steady-state diffusional flux into a partially absorbing body".

1 Implementation of the diffusion simulation

1.1 Overview

In our simulation the geometry of the convex body A is completely specified by two (related) functions. First, a function $d(\mathbf{r},A)$ which gives the signed closest distance of any point $\mathbf{r}\in\mathbb{R}^3$ to the surface of A, with the convention that $d(\mathbf{r},A)>0$ for $\mathbf{r}$ *outside* of A. This function encodes all the information on the shape of A that is required. Second, a function $\mathbf{R}_A(\mathbf{r})$ which yields the point in ∂A closest to $\mathbf{r}$. Note that due to the assumed convexity of A this latter function is uniquely defined. For a sphere these functions are trivial, while for spheroids, they are more involved. In the latter case we employ a computationally convenient analytical lower-bound on the distance. The details on this are provided separately in section 2.

The simulation algorithm itself has only three tuneable parameters: the radius R_B of the enclosing sphere B, a cross-over distance to the surface ∂A denoted by d_*, below which the algorithm switches to a finite time step Brownian simulation with step size $\lambda=\sqrt{4D\Delta t}$, the latter being the final parameter.

The algorithm tracks the fate of individual trial particles. Each particle starts in a randomly selected location $\mathbf{r}_0$ on the surrounding sphere B, in the state we denote by $\mathscr{S}$ (Sphere). It then performs a walk-on-spheres (WoS) move[1] by choosing a random position $\mathbf{r}_1$ on the surface of a sphere $B(d_A(\mathbf{r}_0)|\mathbf{r}_0)$. After this step the particle can be in one of three states

- $\mathscr{I}$ *Interior*: We have $d_A(\mathbf{r}_1)\geq d_*$ *and* $|\mathbf{r}|\leq R_B$. In this case we perform another WoS move by picking a new position $\mathbf{r}_2$ randomly on the surface of the sphere $B(d(\mathbf{r},A)|\mathbf{r}_1)$.

- $\mathscr{O}$ *Outside*: We have $|\mathbf{r}_1|>R_B$. In this case the particle either escapes to infinity, the absorbing state $\mathscr{E}$, or re-enters the enclosing sphere B at a location $\mathbf{r}_2\in\partial B$ returning to state $\mathscr{S}$. Both the probability of escape as well as the probability density for the re-entry point are exactly known, being simply related to the charge density induced on a spherical perfect conductor due to a negative charge located outside of it. We provide the details and we provide the details in section 1.2 below.

- $\mathscr{L}$ *Local*: We have $0\leq d_A(\mathbf{r}_1)<d_*$ In this case a finite time step Brownian algorithm with reflective boundary condition at ∂A is run until the particle exits the boundary layer to a position $\mathbf{r}'$ with $d_A(\mathbf{r}')>d_*$. The details of this algorithm are given in section 1.3 below.

Each trial ends with the particle escapes to infinity into the absorbing state $\mathscr{E}$. This algorithm is than repeated for N times, with N chosen to obtain the desired statistics. The algorithm is schematically illustrated in Figure 1.

1.2 The return to the sphere problem

Consider a particle located at a position $\mathbf{r}$ outside of the enclosing sphere $B=B(\mathbf{0},R_B)$, i.e. $|\mathbf{r}|>R_B$. The probability density of this particle surviving and arriving at $\mathbf{r}'$ without being killed by hitting the sphere at any intermediate time satisfies the

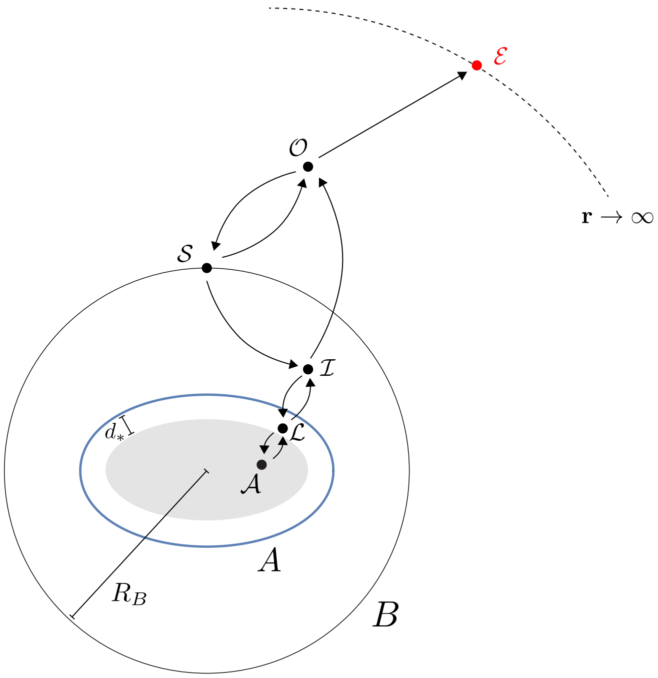

Figure 1. Schematic of the stochastic diffusion algorithm. The particle starts in state $\mathscr{S}$ on the surface of the reference sphere B. In this state, as well as the interior state $\mathscr{I}$ it performs walk-on-sphere moves. In the boundary layer state $\mathscr{L}$ it performs Brownian diffusion with reflection at the surface ∂A whenever it end up inside A (the transient state $\mathscr{A}$). Outside of the reference sphere (state $\mathscr{O}$)) it either returns to the surface of the sphere (state $\mathscr{S}$) or escapes to infinity into the single absorbing state $\mathscr{E}$.

time-dependent problem (see e.g.[2])

$$\frac{\partial}{\partial t}s\left(\mathbf{r}',t|\mathbf{r},0\right)=D\Delta s\left(\mathbf{r}',t|\mathbf{r},0\right) \tag{1}$$

with boundary condition

$$s\left(\left|\mathbf{r}'\right|=R_B,t|\mathbf{r},0\right)=0 \tag{2}$$

and initial condition

$$s\left(\mathbf{r}',0|\mathbf{r},0\right)=\delta\left(\mathbf{r}'-\mathbf{r}\right) \tag{3}$$

The probability flux density of the particle being absorbed at any point $\mathbf{R}$ on the sphere is given by

$$\varphi\left(\mathbf{R},t|\mathbf{r}\right)=D\nabla s\left(\mathbf{R},t|\mathbf{r},0\right)\cdot\hat{\omega}\left(\mathbf{R}\right),\ \mathbf{R}\in\partial B \tag{4}$$

Integrating over all time we find that the ultimate survival probability density satisfies the Poisson equation

$$\Delta S\left(\mathbf{r}'|\mathbf{r}\right)=-\frac{1}{D}\delta\left(\mathbf{r}'-\mathbf{r}\right) \tag{5}$$

with boundary condition

$$S\left(\mathbf{R}|\mathbf{r}\right)=0,\ \mathbf{R}\in\partial B \tag{6}$$

which we recognize as the problem for the electrostatic potential field of a charge $Q=-1/D$ located at $\mathbf{r}$ in the presence of a spherical perfect conductor of radius R_B. The ultimate probability of first being absorbed at $\mathbf{R}\in\partial B$ is then

$$P_B\left(\mathbf{R}|\mathbf{r}\right)=D\nabla S\left(\mathbf{R}|\mathbf{r}\right)\cdot\hat{\omega}\left(\mathbf{R}\right) \tag{7}$$

which is nothing but the charge density on the conducting surface induced by a unit negative charge at $\mathbf{r}$. The solution to this problem using e.g. the method of image charges is textbook material[3]. The result is

$$P_B\left(\mathbf{r}\right)=\frac{R_B}{|\mathbf{r}|}, \tag{8}$$

which is the total charge induced on the conductor. Likewise, the probability density of it returning to a specific position $\mathbf{R}$ on the surface is given by the induced surface charge at that location

$$p_B\left(\mathbf{R}\in\partial B|\mathbf{r}\right)=\frac{1}{4\pi R_B|\mathbf{r}|}\frac{\left(1-\frac{R_B^2}{|\mathbf{r}|^2}\right)}{\left(1-2\frac{R_B}{|\mathbf{r}|}\cos\theta+\frac{R_B^2}{|\mathbf{r}|^2}\right)^{\frac{3}{2}}}\equiv p_B\left(\cos\theta\left|\frac{R_B}{|\mathbf{r}|}\right.\right) \tag{9}$$

where

$$\cos\theta=\frac{\mathbf{R}\cdot\mathbf{r}}{R_B|\mathbf{r}|}. \tag{10}$$

The algorithm thus first determines whether the particle escapes to infinity (probability $1-P_B(\mathbf{r})$), and if not generates a new position $\mathbf{r}'$ on the enclosing sphere by sampling from the distribution (9). The latter is readily done by considering by the cumulative distribution of the polar cosine $\xi=\cos\theta$, which works out as

$$\Pi\left(\xi|P\right)=\int_{-1}^{\xi}d\xi'\,p_B\left(\xi'|P\right)=\frac{1}{2}\left(1-P^2\right)\frac{(1+P)-\left(1-2P\xi+P^2\right)^{\frac{1}{2}}}{P(1+P)(1-2P\xi+P^2)^{\frac{1}{2}}}, \tag{11}$$

where for brevity we have set $P=R_B/|\mathbf{r}|$. Thus choosing ρ uniformly on $[0,1]$ we sample ξ by calculating

$$\xi=\frac{2\rho^2P\left(P^2+1\right)-2\rho\left(P^2+1\right)(P-1)-(P-1)^2}{((2\rho-1)P+1)^2} \tag{12}$$

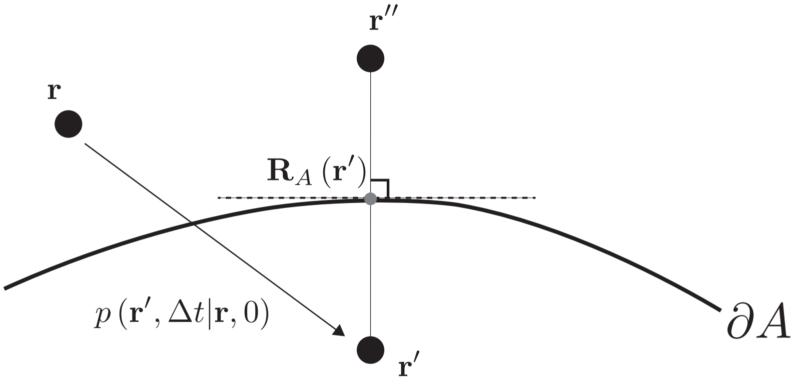

Figure 2. The reflection rule at the surface of body A, showing a transition into an interior location $\mathbf{r}'$ followed by a specular reflection with respect to the tangent plane at the surface point $\mathbf{R}_A(\mathbf{r}')$.

1.3 Brownian diffusion with reflection

Within the boundary layer $L = \{\mathbf{r}|0 \le d(\mathbf{r}, A) < d_*\}$ the particle performs a standard Brownian random walk. Starting from a position $\mathbf{r}$ with $d(\mathbf{r}, A) > 0$ a new position $\mathbf{r}'$ is sampled from the finite time step diffusion probability distribution

$$p(\mathbf{r}', \Delta t|\mathbf{r}, 0) = \frac{1}{(4\pi D\Delta t)^{3/2}} e^{-\frac{(\mathbf{r}'-\mathbf{r})^2}{4D\Delta t}}, \tag{13}$$

where we will use the quantity $\lambda = \sqrt{4D\Delta t}$ as a measure for the step size.

If the chosen location is inside A, i.e. $d(\mathbf{r}', A) < 0$, which we denote as the (transient) state $\mathscr{A}$, we determine the point on the surface $\mathbf{R}_A(\mathbf{r}')$ closest to it and reflect the particle in the tangent plane to the surface at this point

$$\mathbf{r}'' = 2\mathbf{R}_A(\mathbf{r}') - \mathbf{r}'. \tag{14}$$

The relevant geometry is illustrated in Figure 2. This process is repeated until the particle exits the boundary layer and switches to the WoS algorithm.

2 Lower bound on distance to a spheroid

We consider the closest distance $d(\mathbf{r}, A)$ and the closest point on the surface $\mathbf{R}_A(\mathbf{r})$ when A is a spheroid. Consider the defining function of a spheroid

$$F(\mathbf{R}) = \frac{X^2}{a^2} + \frac{Y^2}{a^2} + \frac{Z^2}{b^2} - 1 \equiv \frac{W^2}{a^2} + \frac{Z^2}{b^2} - 1 \tag{15}$$

This function vanishes on the surface of the spheroid, $F(\mathbf{R}) = 0$, $\mathbf{R} = (X, Y, Z) \in \partial A$. It is geometrically obvious that the point on the surface closest an external point $\mathbf{r} = (x, y, z)$ is obtained by dropping a plumb-line from $\mathbf{r}$ to the surface. By construction $\nabla F(\mathbf{R})|_{\mathbf{R}\in\partial A}$ is perpendicular to the surface, allowing us to parametrize

$$\mathbf{r} - \mathbf{R} = \frac{1}{2} t \nabla F(\mathbf{R}) \tag{16}$$

where for notational convenience we have dropped the explicit dependence of $\mathbf{R}$ on $\mathbf{r}$. Eq. (16) is readily solved for $\mathbf{R}(t)$ to yield

$$W^2 = \frac{a^4 w^2}{(t + a^2)^2}, \quad Z^2 = \frac{b^4 z^2}{(t + b^2)^2} \tag{17}$$

where $w^2 = x^2 + y^2$. In order for $\mathbf{R}(t)$ to be on the surface, we require

$$F(t) \equiv F(\mathbf{R}(t)) = \frac{a^2 w^2}{(t + a^2)^2} + \frac{b^2 z^2}{(t + b^2)^2} - 1 = 0 \tag{18}$$

One checks that this function has the following properties: $F\left(t=-\min(a^2,b^2)\right)=+\infty$, $F(t\to\infty)=-1$, $F'(t)<0$ and $F''(t)>0$. The unique real root t_0 on $[-\min(a^2,b^2),\infty)$, where for $\mathbf{r}$ outside A, $t_0>0$, solves our problem as $\mathbf{R}(t_0)$ is the closest point, and $d^2(t_0)$, where

$$d^2(t)=|\mathbf{r}-\mathbf{R}(t)|^2=\frac{t^2w^2}{(t+a^2)^2}+\frac{t^2z^2}{(t+b^2)^2}, \tag{19}$$

provides the smallest distance. However, to obtain t_0 requires solving a fourth order polynomial equation. While this is numerically tractable using an iterative technique such as Newton-Raphson, it is costly to perform in our algorithm where it is required for every WoS move.

Given the convexity of $F(t)$ we know that the curve lies above the tangent line at $t=0$, which is readily obtained by expansion

$$F_1(t)=-2t\left(\frac{w^2}{a^4}+\frac{z^2}{b^4}\right)+\frac{w^2}{a^2}+\frac{z^2}{b^2}-1. \tag{20}$$

The root of $F_1(t)=0$,

$$t_1=\frac{\frac{w^2}{a^2}+\frac{z^2}{b^2}-1}{2\left(\frac{w^2}{a^4}+\frac{z^2}{b^4}\right)}, \tag{21}$$

therefore has the property $t_1<t_0$. Since, as one checks, $d^2(t)$ is concave, it follows that also $d^2(t_1)<d^2(t_0)$. This implies that $d(t_1)$ is a lower bound for the distance to the surface, which can safely be used in the WoS algorithm, trading efficiency in terms of maximizing the jump size for computational efficiency. Note, however, that by construction, the approximation becomes better as the starting point comes closer to the surface, so that overall performance is not too strongly impacted.

3 The absorption probability of a Brownian particle in contact with a partially absorbing smooth convex surface

We require the probability that a particle performing a finite time step diffusive process is absorbed whenever it crosses the boundary a partially absorbing body. This probability has to be chosen such that in the limit of a vanishingly small time step Δt the solution to the diffusion problem formulated in the main text (Eqs. (1) and (2)) with the Robin boundary conditions Eq. (4) is recovered.

The work of Feller[4] had already shown that a one-dimensional partially absorbing diffusion boundary can be constructed by taking the limit of a continuous time Brownian process in which the particle is either absorbed with probability p_a or instantaneously "reflected" to a position ε_a. In this limit we take $p_a\downarrow 0$ and $\varepsilon_a\downarrow 0$ such that the ratio$p_a/\varepsilon_a\to c$, where the constant $c\in[0,\infty)$ governs the degree of absorption, with $c=0$ describing a reflecting boundary and $c\to\infty$ a fully absorbing one. The extension to 3D of Feller's result was later considered by a number of authors (see e.g.,[5,6] and references therein). Here we follow the more recent approach of Singer et al.[7], hereafter referred to as SSOH. They argue that, as the size of a typical spatial step in the finite time step BD simulation scales as $\Delta x\propto\sqrt{\Delta t}$, the approximate absorption probability should therefore also scale as

$$p_a(\Delta t)=P\sqrt{\Delta t}. \tag{22}$$

Note that as Δt approaches zero the absorption probability actually vanishes. The microscopic process therefore *does not* satisfy the partially absorptive boundary condition, because it creates a so-called boundary layer of thickness $\propto\sqrt{\Delta t}$. To match the solution of the microscopic process to the macroscopic diffusion solution requires a boundary layer analysis employing a matched asymptotic expansion, which is described in SSOH for the case of a planar boundary. However, SSOH already remarked that the result should also carry over to the case of a sufficiently smooth curved boundary. Here we explicitly show that this is indeed the case for a smooth convex bounding surface ∂A, provided the local radii of this surface are large compared to the scale of the free diffusion length in a single time step.

To set up the boundary layer analysis one first introduces as small parameter the length $\lambda=\sqrt{4D\Delta t}$. The absorption probability at the location $\mathbf{R}\in\partial A$ is then given by $p_a(\mathbf{R},\lambda)=\rho(\mathbf{R})\lambda$, where $\rho(\mathbf{R})$ needs to be determined. For any position $\mathbf{r}$ outside A we can define $\mathbf{R}_\perp(\mathbf{r})$ as the point closest to $\mathbf{r}$ on the surface of A uniquely due to the convexity of A. Let $n_{\Delta t}(\mathbf{r},t)$ be the density of the Brownian diffusion process, where we suppress the for our purposes irrelevant dependency on initial conditions. The process satisfies the forward Kolmogorov equation

$$n_{\Delta t}(\mathbf{r},t+\Delta t)=\int_{\mathbb{R}^3/A}d\mathbf{r}'\,p_{\Delta t}\left(\mathbf{r},t+\Delta t|\mathbf{r}',t\right)n_{\Delta t}\left(\mathbf{r}',t\right) \tag{23}$$

where the propagator is given by

$$p_{\Delta t}\left(\mathbf{r},t+\Delta t|\mathbf{r}',t\right)=p_0\left(\mathbf{r},t+\Delta t|\mathbf{r}',t\right)+\left(1-\rho\left(\mathbf{R}_\perp(\mathbf{r})\right)\lambda\right)p_0\left(2\mathbf{R}_\perp(\mathbf{r})-\mathbf{r},t+\Delta t|\mathbf{r}',t\right) \tag{24}$$

We now introduce a local coordinate frame (X,Y,Z) with $\mathbf{R}_\perp(\mathbf{r})$ as the origin and

$$\hat{\mathbf{e}}_Z=\hat{\omega}\left(\mathbf{R}_\perp\right)=\frac{\mathbf{r}-\mathbf{R}_\perp(\mathbf{r})}{|\mathbf{r}-\mathbf{R}_\perp(\mathbf{r})|} \tag{25}$$

as the direction of the positive Z-axis. For a sufficiently smooth surface we can then choose two unit tangent vectors $\hat{\mathbf{e}}_X$ and $\hat{\mathbf{e}}_Y$ along the principal directions of curvature such that $\hat{\mathbf{e}}_X\wedge\hat{\mathbf{e}}_Y=\hat{\mathbf{e}}_Z$. The surface is then locally represented by the quadric

$$Z_C(X,Y)=-\frac{1}{R_X}X^2-\frac{1}{R_Y}Y^2+O\left(X^pY^q,p+q=3\right) \tag{26}$$

where R_X and R_Y are the principle radii of curvature at $\mathbf{R}_\perp(\mathbf{r})$. As we will be considering the limit $\lambda\downarrow 0$ it is clear that the dominant contributions to the Kolmogorov integral will come from points which are "close" to the origin. Specifically we can assume that $\lambda\ll R_X,R_Y$. In that case we can write

$$\begin{aligned}
&n_{\Delta t}(0,0,Z,t+\Delta t)=\\
&\int_{-\infty}^{\infty}dX'\int_{-\infty}^{\infty}dY'\int_{Z_C(X',Y')}^{\infty}dZ'\,p_{\Delta t}\left(0,0,Z,t+\Delta t|X',Y',Z',t\right)n_{\Delta t}\left(X',Y',Z',t\right)\\
&=\frac{1}{\pi^{3/2}\lambda^3}\int_{-\infty}^{\infty}dX'\int_{-\infty}^{\infty}dY'\int_{Z_C(X',Y')}^{\infty}dZ'\,n_{\Delta t}\left(X',Y',Z',t\right)\times\\
&\qquad e^{-X'^2/\lambda^2}e^{-Y'^2/\lambda^2}e^{-\left(Z-Z'\right)^2/\lambda^2}+(1-\rho\lambda)\,e^{-X'^2/\lambda^2}e^{-Y'^2/\lambda^2}e^{-\left(Z+Z'\right)^2/\lambda^2}
\end{aligned} \tag{27}$$

where for convenience we have dropped the explicit dependency of $\rho\left(\mathbf{R}_\perp(\mathbf{r})\right)=\rho(0,0,0)$ on the reflection point.

Now introduce a scaled coordinate in the normal direction $Z=\lambda\zeta$. The *inner* solution is then defined through

$$\underline{n}(X,Y,\zeta,t)=n_{\Delta t}(X,Y,\lambda\zeta,t) \tag{28}$$

With this convention, and also scaling the transverse coordinates by setting $X=\lambda\xi$ and $Y=\lambda\eta$, we get the equation

$$\begin{aligned}
\underline{n}\left(0,0,\zeta,t+\frac{\lambda^2}{2D}\right)=\frac{1}{\pi^{3/2}}\int_{-\infty}^{\infty}d\xi'\int_{-\infty}^{\infty}d\eta'\int_{-\lambda\tilde{Z}_C(\xi',\eta')}^{\infty}d\zeta'\underline{n}\left(\lambda\xi',\lambda\eta',\zeta,t\right)\times\\
e^{-\xi'^2}e^{-\eta'^2}\left\{e^{-\left(\zeta-\zeta'\right)^2}+(1-\rho\lambda)\,e^{-\left(\zeta+\zeta'\right)^2}\right\}
\end{aligned}$$

where

$$\tilde{Z}_C(\xi,\eta)=\frac{\xi^2}{R_X}+\frac{\eta^2}{R_Y}. \tag{29}$$

One then assumes that the inner solution has the expansion

$$\underline{n}(X,Y,\zeta,t)=\underline{n}^{(0)}(X,Y,\zeta,t)+\lambda\underline{n}^{(1)}(X,Y,\zeta,t)+O\left(\lambda^2\right). \tag{30}$$

Inserting this expansion and collecting terms of equal order, we find to lowest order

$$\begin{aligned}
\underline{n}^{(0)}(0,0,\zeta,t)&=\frac{1}{\pi^{3/2}}\int_{-\infty}^{\infty}d\xi'\int_{-\infty}^{\infty}d\eta'\int_{0}^{\infty}d\zeta'\,\underline{n}^{(0)}\left(0,0,\zeta',t\right)e^{-\xi'^2}e^{-\eta'^2}\left\{e^{-\left(\zeta-\zeta'\right)^2}+e^{-\left(\zeta+\zeta'\right)^2}\right\}\\
&=\frac{1}{\pi^{1/2}}\int_{-\infty}^{\infty}d\zeta'\,\underline{n}^{(0)}\left(0,0,\zeta',t\right)e^{-\left(\zeta-\zeta'\right)^2}
\end{aligned} \tag{31}$$

which has the obvious solution $\underline{n}^{(0)}(0,0,\zeta,t)=\underline{n}^{(0)}(t)$, i.e. *in*dependent of ζ.

The next order yields

$$\underline{n}^{(1)}(0,0,\zeta,t) = \frac{1}{\pi^{3/2}}\int_{-\infty}^{\infty} d\xi' \int_{-\infty}^{\infty} d\eta' \int_{0}^{\infty} d\zeta' \, \underline{n}^{(1)}\left(0,0,\zeta',t\right) e^{-\xi'^2} e^{-\eta'^2} \left\{ e^{-\left(\zeta-\zeta'\right)^2} + e^{-\left(\zeta+\zeta'\right)^2} \right\} \tag{32}$$
$$+ \frac{1}{\pi^{3/2}}\int_{-\infty}^{\infty} d\xi' \int_{-\infty}^{\infty} d\eta' \int_{0}^{\infty} d\zeta' \, \frac{\partial}{\partial X}\underline{n}^{(0)}\left(0,0,\zeta',t\right) \xi' e^{-\xi'^2} e^{-\eta'^2} \left\{ e^{-\left(\zeta-\zeta'\right)^2} + e^{-\left(\zeta+\zeta'\right)^2} \right\}$$
$$+ \frac{1}{\pi^{3/2}}\int_{-\infty}^{\infty} d\xi' \int_{-\infty}^{\infty} d\eta' \int_{0}^{\infty} d\zeta' \, \frac{\partial}{\partial Y}\underline{n}^{(0)}\left(0,0,\zeta',t\right) e^{-\xi'^2} \eta' e^{-\eta'^2} \left\{ e^{-\left(\zeta-\zeta'\right)^2} + e^{-\left(\zeta+\zeta'\right)^2} \right\}$$
$$- \frac{\rho}{\pi^{3/2}}\int_{-\infty}^{\infty} d\xi' \int_{-\infty}^{\infty} d\eta' \int_{0}^{\infty} d\zeta' \, \underline{n}^{(0)}\left(0,0,\zeta',t\right) e^{-\xi'^2} e^{-\eta'^2} e^{-\left(\zeta+\zeta'\right)^2}$$
$$+ \lim_{\lambda\downarrow 0} \frac{1}{\lambda\pi^{3/2}}\int_{-\infty}^{\infty} d\xi' \int_{-\infty}^{\infty} d\eta' \int_{-\lambda\tilde{Z}_C(\xi',\eta')}^{0} d\zeta' \, \underline{n}^{(0)}\left(0,0,\zeta',t\right) e^{-\xi'^2} e^{-\eta'^2} \left\{ e^{-\left(\zeta-\zeta'\right)^2} + e^{-\left(\zeta+\zeta'\right)^2} \right\}$$

The second and third terms vanish by symmetry. The last term also vanishes since

$$\int_{-\lambda\tilde{Z}_C(\xi',\eta')}^{0} d\zeta' \left\{ e^{-\left(\zeta-\zeta'\right)^2} + e^{-\left(\zeta+\zeta'\right)^2} \right\} = 2\lambda^2 \tilde{Z}_C\left(\xi',\eta'\right)^2 \zeta e^{-\zeta^2} + O\left(\lambda^3\right) \tag{33}$$

Using the result

$$\int_{0}^{\infty} d\zeta' \, e^{-\left(\zeta+\zeta'\right)^2} = \frac{\sqrt{\pi}}{2}\operatorname{erfc}(\zeta) \tag{34}$$

the remaining two terms are then readily evaluated to yield

$$\underline{n}^{(1)}(0,0,\zeta,t) = \frac{1}{\pi^{1/2}}\int_{0}^{\infty} d\zeta' \, \underline{n}^{(1)}\left(0,0,\zeta',t\right) \left\{ e^{-\left(\zeta-\zeta'\right)^2} + e^{-\left(\zeta+\zeta'\right)^2} \right\}$$
$$- \frac{1}{2}\rho \operatorname{erfc}(\zeta)\, \underline{n}^{(0)}(t). \tag{35}$$

This shows that to first order in λ the geometry of the surface does not affect the expansion of the inner solution in the perpendicular direction. As by construction the expansion of the *outer* solution

$$n_{\Delta t}(X,Y,Z,t) = n^{(0)}(X,Y,Z,t) + \lambda n^{(1)}(X,Y,Z,t) + \ldots \tag{36}$$

is local in the transverse coordinates X, Y, the remaining analysis is identical to the one given by SSOH. The end result thus is the one found in Eq. (1.14) of SSOH

$$P = \frac{\sqrt{\pi}}{\sqrt{D}}k. \tag{37}$$

4 Markov chain analysis of the steady-state absorption probabilities

The analysis of Markov chains with absorbing states is standard (see e.g. the textbook by Grinstead & Snell[8]). We distinguish the non-absorbing states $(0,1,2,\ldots,M)$ and the absorbing states $(\infty,1^*,2^*,\ldots,M^*)$. The transition matrix between the transient non-absorbing states is given by

$$\mathbf{T} = \begin{bmatrix} 0 & p_{i,1} & \cdots & p_{i,M} \\ 0 & q_1^* p_{1,1} & \cdots & q_1^* p_{1,M} \\ 0 & q_2^* p_{2,1} & \cdots & q_2^* p_{2,M} \\ \vdots & & \cdots & \\ 0 & q_M^* p_{M,1} & \cdots & q_M^* p_{M,M} \end{bmatrix} \tag{38}$$

The matrix for the transitions from the transient states to the absorptive states is given by

$$\mathbf{A} = \begin{bmatrix} q_i^* p_{i,\infty} & 0 & \cdots & 0 \\ q_1^* p_{1,\infty} & p_1^* & \cdots & 0 \\ \vdots & & \ddots & \\ q_1^* p_{M,\infty} & 0 & \cdots & p_M^* \end{bmatrix} \tag{39}$$

The full transition matrix describing of the system is

$$\mathbf{F} = \begin{bmatrix} \mathbf{T} & \mathbf{A} \\ \mathbf{0} & \mathbf{I} \end{bmatrix} \tag{40}$$

To calculate the probability of absorption at each absorptive state we first calculate the fundamental matrix **N**

$$\mathbf{N} = (\mathbf{I} - \mathbf{T})^{-1} \tag{41}$$

The (i, j) entry of **N** is the expected number of times the particles visit a state j, given that the they started in state i, before arriving at an absorbing state (either within a surface domain m or by escaping to ∞). Similarly the absorption probabilities are given by the entries of the following matrix

$$\mathbf{B} = \mathbf{N}\mathbf{A}, \tag{42}$$

where the entry (i, j) of **B** is the probability of absorption starting from the transient state i to the absorbing state j^*. Since all the particles start from the reference sphere (state 0) we are interested only in the first row of **B**. The probability of absorption to a state i is starting from the initial state is given by

$$P_i^* = B_{0,i} \tag{43}$$

The steady-state flux into the surface domain i is then

$$\Phi_i = B_{0,i}\Phi_{\text{in}} \tag{44}$$

where Φ_{in} is either the flux into the reference sphere due to the source at infinity Φ_B or the flux of the fixed point source Φ_S (see section Problem Formulation of the main text). The coarse grained flux density within domain i is then estimated as

$$\varphi_i = M \frac{B_{0,i}}{|\partial A|}\Phi_{\text{in}} \tag{45}$$

where $|\partial A|$ is the surface area of the body A. Finally, the total flux through the body is estimated as

$$\Phi_A = \sum_{i=1}^{M} B_{0,i}\Phi_{\text{in}} = (1 - B_{0,0})\Phi_{\text{in}}. \tag{46}$$

5 Approximate solutions for spheroidal bodies

5.1 Spheroid geometry

A prolate spheroid is the 3D shape obtained by rotating an ellipse around its longest axis. A 3D coordinate system which is adapted to this geometry is given by the so-called prolate spheroidal coordinates, which are defined defined through the parametrization of a point $\mathbf{r} = (x, y, z) \in \mathbb{R}^3$ through

$$\mathbf{r} = (L \sinh\alpha \sin\beta \cos\varphi, L \sinh\alpha \sin\beta \sin\varphi, L \cosh\alpha \cos\beta) \tag{47}$$

where $\alpha \in [0, \infty), \beta \in [0, \pi)$ and $\varphi \in [0, 2\pi)$. We see that

$$\frac{x^2 + y^2}{L^2 \sinh^2\alpha} + \frac{z^2}{L^2 \cosh^2\alpha} = 1 \tag{48}$$

so that the “equipotential” surface at a fixed value α_0 is that of an ellipsoid of revolution with (semi) major axis length $c = L\cosh\alpha_0$ and equal (semi)minor axis lengths $a = b = L\sinh\alpha_0$, and hence aspect ratio $\gamma \equiv c/a = \coth\alpha_0$.

5.2 Two parameter family of inhomogeneous absorption rate distributions

As the angle φ describes the azimuthal angle corresponding to rotations around the long axis of the spheroid, an axially symmetric distribution of local absorption rates on the surface of a spheroid only depends on the angle β, or equivalently $\xi = \cos\beta$. A simple non-trivial distribution which is able to describe both unipolar, bipolar and mixed distributions has the form

$$\kappa(\xi) = \kappa_0 + \kappa_1 P_1(\xi) + \kappa_2 P_2(\xi), \tag{49}$$

where $P_l(\xi)$ are Legendre polynomials and $\kappa(\xi) = k(\xi)/\bar{k}$ the dimensionless local absorption rate. However, in order to be an acceptable distribution we must first require $\kappa(\xi) \geq 0$. It is clear that $\kappa_0 > 0$ and we conveniently define $\tilde{\kappa}_i = \kappa_i/\kappa_0$. An analysis of the roots of (49) reveals that this requires

$$\tilde{\kappa}_1 \in \begin{cases} [-1-\tilde{\kappa}_2, 1+\tilde{\kappa}_2] & -1 \leq \tilde{\kappa}_2 \leq \frac{1}{2} \\ [-\sqrt{6\tilde{\kappa}_2(1-\frac{1}{2}\tilde{\kappa}_2)}, \sqrt{6\tilde{\kappa}_2(1-\frac{1}{2}\tilde{\kappa}_2)}] & \frac{1}{2} \leq \tilde{\kappa}_2 \leq 2 \end{cases} \tag{50}$$

which is an ‘ice-cream cone’-like region in $(\tilde{\kappa}_1, \tilde{\kappa}_2)$ space. Secondly, as we condition on the mean absorption rate, we must also fix

$$\frac{\int_{\partial A} d\sigma(\alpha_0,\beta,\varphi)\,\kappa(\cos\beta)}{\int_{\partial A} d\sigma(\alpha_0,\beta,\varphi)} = 1, \tag{51}$$

where $d\sigma(\alpha_0,\beta,\varphi) = L^2 \sinh\alpha_0 \sqrt{\cosh^2\alpha_0 - \cos^2\beta}\,\sin\beta\, d\beta\, d\varphi$ is the element of surface area on the spheroid. Defining the family of integrals

$$S_l = \int_{\partial C} d\sigma(\alpha_0,\beta,\varphi)\, P_l(\cos\beta) = 2\pi L^2 \sinh\alpha_0 \int_{-1}^{1} d\xi \sqrt{\cosh^2\alpha_0 - \xi^2} P_l(\xi), \tag{52}$$

the relevant ones are

$$S_0 = 2\pi L^2 \sinh\alpha_0 \left(\sinh\alpha_0 + \cosh^2\alpha_0 \arcsin\frac{1}{\cosh\alpha_0}\right) = |\partial A| \tag{53}$$

$$S_1 = 0 \tag{54}$$

$$S_2 = 2\pi L^2 \sinh\alpha_0 \left(\frac{1}{8}\left(\left(2-3\cosh^2\alpha_0\right)\sinh\alpha_0 + \left(3\cosh^2\alpha_0 - 4\right)\cosh^2\alpha_0 \arcsin\frac{1}{\cosh\alpha_0}\right)\right) \tag{55}$$

The constraint thus becomes

$$\int_{\partial C} d\sigma(\alpha_0,\beta,\varphi)\,\kappa(\cos\beta) = \kappa_0 S_0 + \kappa_2 S_2 = S_0, \tag{56}$$

from which we deduce $\kappa_0 \in [S_0/(S_0 - S_2), S_0/(S_0 + 2S_2)]$, where we have used the fact that $-1 \leq S_2 \leq 0$ and the constraint $-\kappa_0 \leq \kappa_2 \leq 2\kappa_0$ derived above.

5.3 The Piazza-Grebenkov formalism

The Piazza-Grebenkov formalism starts from the general solution of the Poisson equation with fixed boundary condition at infinity in spheroiodal coordinates

$$n(\cosh\alpha,\beta) = n_\infty + \sum_{l=0} n_l Q_l(\cosh\alpha)\, P_l(\cos\beta), \tag{57}$$

where the Legendre function of the second kind is explicitly given by

$$Q_l(z) = 2^{-(l+1)}\pi^{1/2}\frac{\Gamma(l+1)}{\Gamma\left(l+\frac{3}{2}\right)} z^{-(l+1)}{}_2F_1\left(1+\frac{l}{2}, \frac{1}{2}+\frac{l}{2}; l+\frac{3}{2}; z^{-2}\right), \; |z| > 1. \tag{58}$$

The boundary condition at the cell surface reads

$$\nabla n(\cosh\alpha_0,\beta)\cdot\hat{\omega}(\alpha_0,\beta) = \frac{1}{L\sqrt{\cosh^2\alpha_0 - \cos^2\beta}}\frac{\partial}{\partial\alpha} n(\cosh\alpha_0,\beta) = \kappa(\cos\beta)\, n(\cosh\alpha_0,\beta). \tag{59}$$

Upon substituting the expansion (57) into this boundary condition, and projecting onto the Legendre polynomials, one obtains an infinite set of linear equation for the unknown expansion coefficients n_l. Recalling the definition $\xi = \cos\beta$ we have, for $l = 0, 1, \ldots, \infty$

$$\frac{1}{L}\sinh\alpha_0 \sum_{l=0} n_l Q_l'(\cosh\alpha_0) \int_{-1}^{1} d\xi \frac{P_{l''}(\xi) P_l(\xi)}{\sqrt{\cosh^2\alpha_0 - \xi^2}} = n_\infty \sum_{l'=0}^{2} \frac{2}{2l'+1} \kappa_{l'} \delta_{l',l''} + \sum_{l'=0}^{2} \sum_{l=0} \kappa_{l'} n_l Q_l(\cosh\alpha_0) \int_{-1}^{1} d\xi P_{l''}(\xi) P_{l'}(\xi) P_l(\xi). \quad (60)$$

The integral over the triple products of Legendre polynomials can be expressed in terms of Wigner 3J-symbols[9]

$$T(l'', l', l) \equiv \int_{-1}^{1} d\xi P_{l''}(\xi) P_{l'}(\xi) P_l(\xi) = 2 \begin{pmatrix} l'' & l' & l \\ 0 & 0 & 0 \end{pmatrix}^2. \quad (61)$$

The integrals

$$F_{l'',l'}(\cosh\alpha_0) = \int_{-1}^{1} d\xi \frac{P_{l''}(\xi) P_{l'}(\xi)}{\sqrt{\cosh^2\alpha_0 - \xi^2}} \quad (62)$$

can be dealt with using the product rules for Legendre polynomials

$$P_{l''}(\xi) P_{l'}(\xi) = \sum_l (2l+1) \begin{pmatrix} l'' & l' & l \\ 0 & 0 & 0 \end{pmatrix}^2 P_l(\xi) \quad (63)$$

and the consideration of the integrals

$$V_l(\cosh\alpha_0) = \int_{-1}^{1} d\xi \frac{P_l(\xi)}{\sqrt{\cosh^2\alpha_0 - \xi^2}}, \quad (64)$$

which PG have shown how these integrals can be recursively calculated. In the following we set $\zeta = \cosh\alpha_0$, and $\sinh\alpha_0 = \sqrt{\zeta^2 - 1}$ so that the set of equations is more compactly stated as

$$\frac{1}{L}\sqrt{\zeta^2 - 1} \sum_{l''=0}^{\infty} n_{l''} Q_{l''}'(\zeta) F_{l,l''}(\zeta) = n_\infty \sum_{l'=0}^{2} \frac{2}{2l'+1} \kappa_{l'} \delta_{l,l'} + \sum_{l'=0}^{2} \sum_{l''=0}^{\infty} \kappa_{l'} n_{l''} Q_{l''}(\zeta) T(l, l', l''). \quad (65)$$

In practice this set is then truncated at a maximal value $l_{\max}$ for the order of the Legendre polynomials included.

5.4 Approximate optimal distribution of absorption rates

We optimize the total flux over the two parameter family of absorption rate distributions defined above using brute force minimization over a grid of parameters. The results are shown in Figure 3. Although by construction the the distributions described by Eq. (49) are less able to focus on towards the poles due to the ‘broadness’ of the first two Legendre polynomials, we do obtain a small, but systematic, gain in the order of 1%.

6 Spherocylinder with a bulk source

We consider spherocylinders comprised of a right circular cylinder of length L and radius R capped at both ends by a half sphere of radius R. The aspect ratio of the cylinder is defined through $\gamma = (L+2R)/2R$. We also employ a scaled longitudinal coordinate along the symmetry axis of the body defined through $\zeta = 2z/(L+2R) \in [-1, 1]$. The surface area is normalized by choosing $2\pi RL + 4\pi R^2 = 4\pi$ (see main text). Note that for a spherocylinder A the nearest distance function $d(\mathbf{r}, A)$ and nearest point function $\mathbf{R}_A(\mathbf{r})$ are geometrically trivial. The results for optimising the local absorption rate distribution are shown in Figure 4. We see that the results are similar to those of the ellipsoids shown in main text Figure 3, but in this case the maximal gain is even below 1% and quickly saturates with increasing aspect ratio.

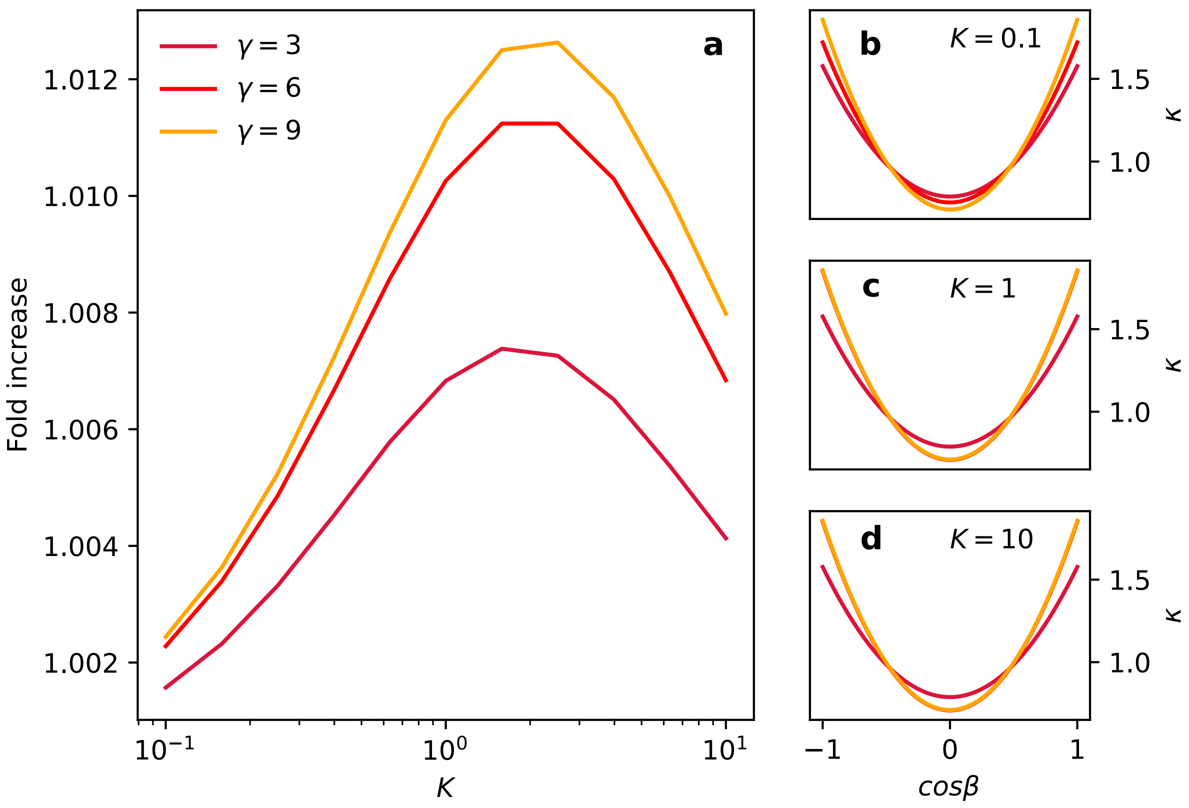

Figure 3. Panel **a**: Fold increase of the total flux with respect to a homogeneously absorbing spheroid as function of the mean absorption rate K, for spheroids with increasing aspect ratio γ using the semi-analytical PG formalism applied to the two-parameter family if absorption rate distributions described by Eq. (49). Panels **b, c, d**: the optimal local absorption rate $\kappa(\cos\beta)$ as function of position parametrized by the polar spheroidal angle β, for three values of the mean absorption rate.

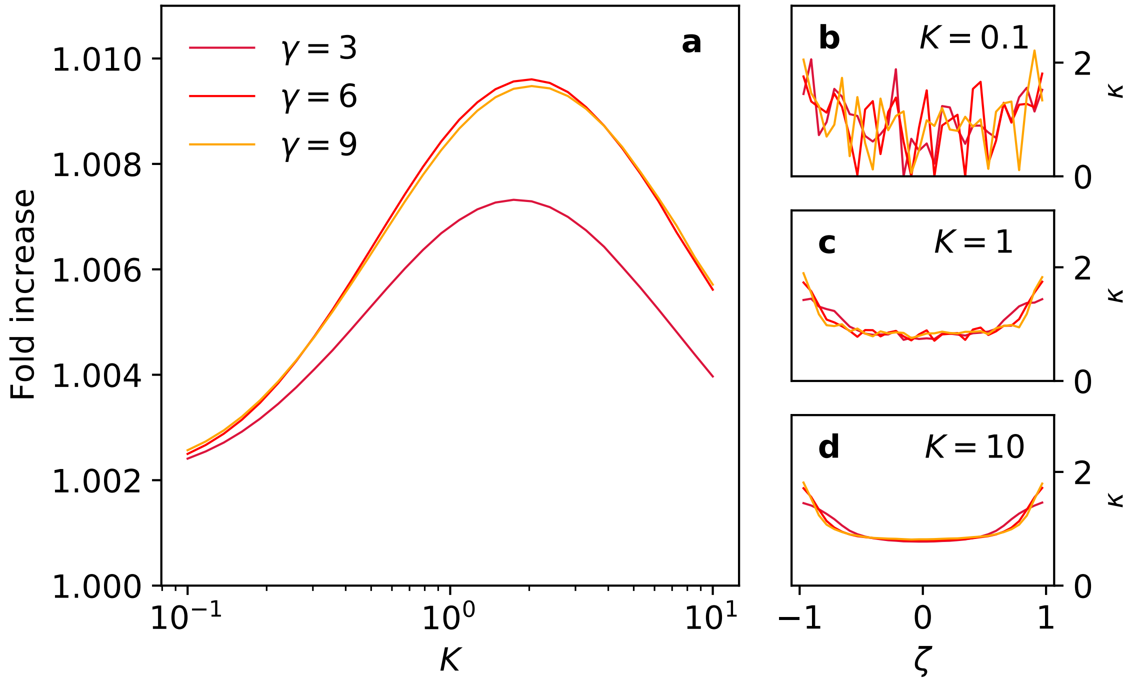

Figure 4. Panel **a**: Fold increase of the total flux with respect to a homogeneously absorbing spherocylinder as function of the mean absorption rate K, for spherocylinders with increasing aspect ratio γ. Panels **b, c, d**: the optimal local absorption rate $\kappa(\zeta)$ as function of reduced longitudinal position ζ for three values of the mean absorption rate.

7 Toy model of two homogeneous partially absorbing spheres

Consider two spheres with radii $R_1 \leq R_2$ that are homogeneously partially absorbing with local absorption rate constants k_1 and k_2 respectively in the presence of a bulk source with value n_∞ at infinity. If the spheres are far enough apart we can approximate the total absorptive flux as the sum of the that of the individual spheres, i.e.

$$\Phi(k_1, k_2) = \Phi_1(k_1) + \Phi_2(k_2), \tag{66}$$

where (see main text Eq. (14))

$$\Phi_i(k_i) = 4\pi D n_\infty R_i \left(\frac{\frac{k_i R_i}{D}}{1 + \frac{k_i R_i}{D}} \right). \tag{67}$$

Let $A_i = 4\pi R_i^2$ be the areas of the spheres. We now impose that the mean absorption rate is fixed

$$\frac{A_1}{A_1 + A_2} k_1 + \frac{A_2}{A_1 + A_2} k_2 = \bar{k}. \tag{68}$$

It is now convenient to take R_1 as our unit of length, and introduce the size ratio $\rho = R_2/R_1 \geq 1$. The area fractions are then expressed as $\alpha_1 = A_1/(A_1 + A_2) = 1/(1+\rho^2)$ and $\alpha_2 = 1 - \alpha_1$. We define the dimensionless absorption rates through $\kappa_i = k_i/\bar{k}$, so that the constraint becomes $\alpha_1\kappa_1 + \alpha_2\kappa_2 = 1$. Note that this implies that $\kappa_1 \in [0, 1/\alpha_1]$.

Setting $K = \bar{k}R_1/D$, and introducing

$$F(\lambda) = \frac{\lambda K}{1 + \lambda K} \tag{69}$$

we can then define our object function as the flux relative to the case where the absorption rate on both spheres is equal

$$\varphi(\kappa_1; K, \rho) = \frac{\Phi(k_1, k_2)}{\Phi(\bar{k}, \bar{k})} = \frac{F(\kappa_1) + \rho F(\rho \frac{1-\alpha_1\kappa_1}{1-\alpha_1})}{F(1) + \rho F(\rho)}. \tag{70}$$

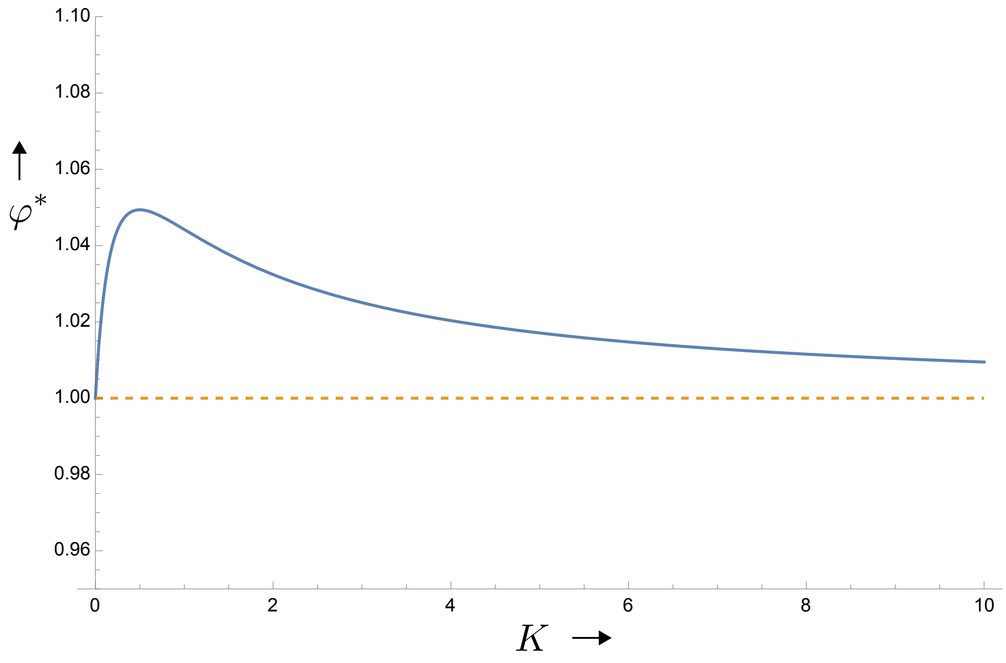

Figure 5. Optimal relative total flux $\varphi(\kappa_1^*, K, 4)$ as a function of the mean absorption rate K for $\rho = 4$.

Maximizing with respect to κ_1 yields the optimum

$$\kappa_1^*(K,\rho) = \frac{1+\rho^2}{1+\rho} \le \frac{1}{\alpha_1} \tag{71}$$

$$\varphi(\kappa_1^*; K,\rho) = \frac{(K+1)(\rho+1)\left(\rho^2+1\right)(K\rho+1)}{(K(\rho^2+1)+(\rho+1))\left((\rho^2+1)+K\rho(\rho+1)\right)} \tag{72}$$

One readily checks that as expected $\varphi(\kappa_1^*, 0, \rho) = \varphi(\kappa_1^*, \infty, \rho) = 1$. From Figure 5 we can already see that the maximal gain is in the order of 5%. To determine the absolute maximum of the gain we first optimize with respect to K yielding

$$K^*(\rho) = \rho^{-\frac{1}{2}} \tag{73}$$

$$\varphi(\kappa_1^*; K^*, \rho) = \frac{(\rho+1)\left(\rho^2+1\right)}{\left(\rho^{3/2}+1\right)^2}, \tag{74}$$

and finally with respect ρ, yielding

$$\rho^* = 1+\sqrt{3}+\sqrt{3+2\sqrt{3}} \simeq 5.27451 \tag{75}$$

$$\varphi(\kappa_1^*; K^*, \rho^*) = \frac{2}{9}\left(3+\sqrt{3}\right) \simeq 1.05157. \tag{76}$$

The fact that the maximal gain is indeed only slightly above 5% nicely illustrates that the gain obtained by focusing the absorption on the smaller sphere is largely offset by the loss of absorption on the larger sphere, due to its larger surface area.

References

1. Muller, M. E. Some Continuous Monte Carlo Methods for the Dirichlet Problem. *The Annals Math. Stat.* **27**, 569–589, DOI: 10.1214/aoms/1177728169 (1956).
2. Redner, S. *A guide to first-passage processes* (Cambridge University Press, Cambridge, 2001).
3. Jackson, J. D. *Classical electrodynamics* (Wiley, 1999).

4. Feller, W. Diffusion Processes in One Dimension. *Transactions Am. Math. Soc.* **77**, 1, DOI: 10.2307/1990677 (1954).

5. Grebenkov, D. S., Filoche, M. & Sapoval, B. Spectral properties of the Brownian self-transport operator. *The Eur. Phys. J. B - Condens. Matter Complex Syst. 2003 36:2* **36**, 221–231, DOI: 10.1140/EPJB/E2003-00339-4 (2003).

6. Grebenkov, D. S. Partially Reflected Brownian Motion: A Stochastic Approach to Transport Phenomena. In Velle, L. R. (ed.) *Focus on Prabability Theory*, chap. 8 (Nova Science Publishers, 2006).

7. Singer, A., Schuss, Z., Osipov, A. & Holcman, D. Partially reflected diffusion. *SIAM J. on Appl. Math.* **68**, 844–868, DOI: 10.1137/060663258 (2007).

8. Grinstead, C. M. & Snell, J. L. *Introduction to probability* (American Mathematical Society, 1997), 2nd edn.

9. Brink, D. M. D. M. & Satchler, G. R. G. R. *Angular momentum* (Clarendon Press, 1994).
